# Genome-wide transcriptome analysis reveals key regulatory networks and genes involved in the determination of seed hardness in vegetable soybean

**DOI:** 10.1093/hr/uhae084

**Published:** 2024-04-02

**Authors:** Congcong Wang, Jianyu Lin, Yuanpeng Bu, Ruidong Sun, Yang Lu, JunYi Gai, Han Xing, Na Guo, Jinming Zhao

**Affiliations:** Key Laboratory of Biology and Genetics Improvement of Soybean, Ministry of Agriculture / Zhongshan Biological Breeding Laboratory (ZSBBL) / National Innovation Platform for Soybean Breeding and Industry-Education Integration / State Key Laboratory of Crop Genetics & Germplasm Enhancement and Utilization / College of Agriculture, Nanjing Agricultural University, Nanjing 210095, China; Key Laboratory of Biology and Genetics Improvement of Soybean, Ministry of Agriculture / Zhongshan Biological Breeding Laboratory (ZSBBL) / National Innovation Platform for Soybean Breeding and Industry-Education Integration / State Key Laboratory of Crop Genetics & Germplasm Enhancement and Utilization / College of Agriculture, Nanjing Agricultural University, Nanjing 210095, China; Key Laboratory of Biology and Genetics Improvement of Soybean, Ministry of Agriculture / Zhongshan Biological Breeding Laboratory (ZSBBL) / National Innovation Platform for Soybean Breeding and Industry-Education Integration / State Key Laboratory of Crop Genetics & Germplasm Enhancement and Utilization / College of Agriculture, Nanjing Agricultural University, Nanjing 210095, China; Key Laboratory of Biology and Genetics Improvement of Soybean, Ministry of Agriculture / Zhongshan Biological Breeding Laboratory (ZSBBL) / National Innovation Platform for Soybean Breeding and Industry-Education Integration / State Key Laboratory of Crop Genetics & Germplasm Enhancement and Utilization / College of Agriculture, Nanjing Agricultural University, Nanjing 210095, China; Key Laboratory of Biology and Genetics Improvement of Soybean, Ministry of Agriculture / Zhongshan Biological Breeding Laboratory (ZSBBL) / National Innovation Platform for Soybean Breeding and Industry-Education Integration / State Key Laboratory of Crop Genetics & Germplasm Enhancement and Utilization / College of Agriculture, Nanjing Agricultural University, Nanjing 210095, China; Key Laboratory of Biology and Genetics Improvement of Soybean, Ministry of Agriculture / Zhongshan Biological Breeding Laboratory (ZSBBL) / National Innovation Platform for Soybean Breeding and Industry-Education Integration / State Key Laboratory of Crop Genetics & Germplasm Enhancement and Utilization / College of Agriculture, Nanjing Agricultural University, Nanjing 210095, China; Key Laboratory of Biology and Genetics Improvement of Soybean, Ministry of Agriculture / Zhongshan Biological Breeding Laboratory (ZSBBL) / National Innovation Platform for Soybean Breeding and Industry-Education Integration / State Key Laboratory of Crop Genetics & Germplasm Enhancement and Utilization / College of Agriculture, Nanjing Agricultural University, Nanjing 210095, China; Key Laboratory of Biology and Genetics Improvement of Soybean, Ministry of Agriculture / Zhongshan Biological Breeding Laboratory (ZSBBL) / National Innovation Platform for Soybean Breeding and Industry-Education Integration / State Key Laboratory of Crop Genetics & Germplasm Enhancement and Utilization / College of Agriculture, Nanjing Agricultural University, Nanjing 210095, China; Key Laboratory of Biology and Genetics Improvement of Soybean, Ministry of Agriculture / Zhongshan Biological Breeding Laboratory (ZSBBL) / National Innovation Platform for Soybean Breeding and Industry-Education Integration / State Key Laboratory of Crop Genetics & Germplasm Enhancement and Utilization / College of Agriculture, Nanjing Agricultural University, Nanjing 210095, China

## Abstract

Seed hardness is an important quality trait of vegetable soybean. To determine the factors underlying seed hardness, two landraces with contrasting seed hardness, Niumaohuang (low seed hardness) and Pixiansilicao (high seed hardness), were selected from 216 soybean accessions originating from 26 provinces in China. The contents of the main components in vegetable soybean seeds such as water, soluble sugar, starch, protein and oil were measured, and transcriptome analyses performed during five stages of seed developmental. Transcriptome analysis indicates that during the middle and late stages of seed development, a large number of genes involved in the synthesis or degradation of starch, storage protein, and fatty acids were differentially expressed, leading to differences in the accumulation of stored substances during seed maturation among Niumaohuang and Pixiansilicao. The activity of cell proliferation and the formation of cell walls in the middle and late stages of seed development may also affect the hardness of seeds to a certain extent. In addition, weighted gene co-expression network analysis (WGCNA) was undertaken to identify co-expressed gene modules and hub genes that regulate seed hardness. Overexpression of a candidate seed hardness regulatory hub gene, *GmSWEET2*, resulted in increased seed hardness. In this study, the important role of *GmSWEET2* in regulating the hardness of vegetable soybean seeds was verified and numerous potential key regulators controlling seed hardness and the proportion of seed components were identified, laying the groundwork for improving the texture of vegetable soybean.

## Introduction

Vegetable soybean [*Glycine max* (L.) Merr.] is the general term for soybeans harvested as vegetable when the seeds are filled with 80% to 90% of the pod width, and the pods and seeds are bright green in color [1]. Vegetable soybean has become a popular vegetable food in Asia due to its delicate taste, aromatic flavor, and high nutritional value. Seed texture is an important factor in sensory evaluation of vegetable soybean, which can affect the chewiness and palatability of vegetable soybean. Consumers in different regions have different preferences for the texture of vegetable soybean. Chinese consumers prefer varieties with soft texture [2]. African consumers prefer varieties with moderate hardness, while varieties with a long chewing time are not popular [3]. Japanese consumers are more receptive to vegetable soybeans with a crisp texture, and US consumers seem to prefer vegetable soybeans with a ‘butter-like’ texture and flavor [4]. In general, excessively hard texture and difficulty in chewing often reduce consumer acceptance of vegetable soybean.

Currently, studies on the hardness of soybean seeds are mostly focused on mature seeds, and the hardness of mature soybeans exerts a significant influence on their edibility and processing characteristics [5]. Previous studies confirmed that there is a negative correlation between the hardness and size of soybean seeds [6, 7]. Soybean seed hardness is affected by the proportion of seed components such as oil and protein [8, 9]. Water absorption [6] and contents of trace elements such as Ca and Mg [10, 11] also affect the hardness of cooked mature soybean seeds. In addition, flavonoids and cell wall components such as lignin, pectin, and hemicellulose have also been verified to affect the hardness of soybean seeds [12–14].

The development and maturation of soybean can be divided into vegetative growth stages (V) and reproductive growth stages (R). Reproductive growth stages consist of eight subdivisions, which are named numerically as R1 (beginning bloom), R2 (full bloom), R3 (beginning pod), R4 (full pod), R5 (beginning seed), R6 (full seed), R7 (beginning maturity), and R8 (full maturity) [15]. Vegetable soybeans were harvested when they were immature, between the reproductive stages of R6 and R7. However, there are relatively few studies on the seed hardness of the harvest stage of vegetable soybean. Previous studies have shown that the seed hardness of vegetable soybean at the R6 stage was negatively correlated with the contents of water, starch, sugar, and seed size, while it was positively correlated with the protein content [3, 16, 17]. In recent studies, a few QTLs and candidate genes involved in regulation of vegetable soybean seed hardness have been identified via conditional and unconditional QTL analyses and association analysis studies [17, 18].

Compared to other crops such as wheat and rice, the research into soybean seed hardness is in its infancy, especially with reference to vegetable soybean. At present, studies on vegetable soybean seed hardness focus only on relatively macroscopic sensory evaluation and influencing factors. Elucidation of the regulatory mechanism of vegetable soybean seed hardness warrants in-depth research. In the present study, we conducted an RNA-Seq analysis for seeds from five developmental stages in two soybean varieties with stable, yet different, seed-hardness phenotypes. Using pairwise comparisons and weighted gene co-expression network analysis (WGCNA), the underlying molecular mechanism of seed hardness difference at the transcriptional level was revealed. We further generated overexpression lines and knockout mutants of a candidate seed hardness regulatory gene, *GmSWEET2*, in soybean. The overexpression lines changed the proportion of different seed substance components and reduced the seed hardness at the R6 stage, as predicted. The results provide a new insight into the molecular network of soybean seed development and the factors determining the seed hardness of vegetable soybean.

## Results

### Evaluation of seed hardness in 216 soybean accessions

To screen vegetable soybean varieties with different seed hardnesses that cater to consumer preferences in different regions, seed hardness of 216 cultivars and landraces from 26 provinces in China could be measured at the R6 stage for three years. The mechanical work (*W*) measured during the puncture process of the texture analyser probe was used to evaluate the hardness of the seeds; *W* (g.mm) ranged from 581.3 to 1835.2, 615.8 to 1647.0, and 549.0 to 1123.8, in 2014, 2015, and 2016, respectively (Fig. S1 and Table S1, see online supplementary material). These results indicate that there are obvious differences in seed hardness among different soybean varieties at the R6 stage. Based on the average value of three-year phenotype data, 46 accessions showed high seed hardness (W > 1000 g.mm) and 36 accessions showed low seed hardness (W < 800 g.mm); the others exhibited intermediate seed hardness (800 g.mm < W < 1000 g.mm).

### Accumulation of substance components related to seed hardness of two soybean landraces with contrasting seed hardness

**Figure 1:**
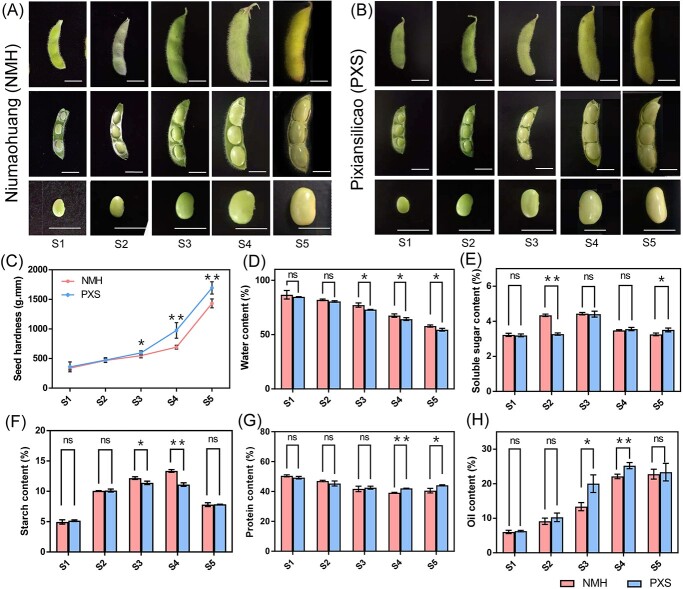
Seed hardness and component contents of NMH and PXS at different stages. (**A**, **B**) Pods and seeds at different stages (S1–S5) of development in NMH and PXS. Bars = 10 mm. (**C**) Seed hardness of NMH and PXS at different stages (expressed as the mechanical work measured during the puncturing). Data are presented as mean ± SD (*n* = 8 biological replicates). ^*^P < 0.05, ^**^P < 0.01, Student’s *t*-test. (**D**–**H**) Water, soluble sugar, starch, protein, and oil contents in NMH and PXS at Stages S1–S5. Data are presented as mean ± SD (*n* = 3 biological replicates). ns, no significant difference. ^*^P < 0.05, ^**^P < 0.01, Student’s *t*-test.

Among 216 soybean accessions, two landraces with stable difference in seed hardness at the R6 stage in multiple environments were selected, namely Niumaohuang (low seed hardness) and Pixiansilicao (high seed hardness), and were respectively referred to as NMH and PXS. In this study, seed development was divided into Stages S1–S5, wherein Stages S1–S3 represented the late stage of beginning seed (R5), Stage S4 corresponded to the full seed stage (R6) and Stage S5 represented the onset of maturity stage (R7, Fig. 1A and B). At Stages S1 and S2, the cotyledons were undergoing filling with storage products, but water accounts for more than 80% of the seed weight (Fig. 1D), so NMH and PXS have the same soft texture at these stages. Significant difference in seed hardness between NMH and PXS first appeared at Stage S3 and became more significant at Stage S4 (Fig. 1C). To evaluate the relationship between the seed hardness and the accumulation of seed components, we measured the contents of the main components in vegetable soybean seeds, including water, soluble sugar, starch, protein, and oil (Fig. 1D–H). In both NMH and PXS, the water content decreased with seed development, and in the last three stages, the water content of NMH was significantly higher than PXS. The soluble sugar content increased and then decreased in both NMH and PXS, peaking at Stage S3, then decreasing, and almost levelling off at Stages S4 and S5. At Stage S2, the soluble sugar content in NMH was significantly higher than that of PXS, but at Stage S5 it was the opposite. Similar to the soluble sugar, the starch content also increased first and then decreased, reaching its peak at Stage S3 or S4, and then decreased significantly at Stage S5. At Stages S3 and S4, the starch content in NMH was significantly higher than that of PXS. The oil content gradually increased with the development of seeds before Stage S4. Significant difference was observed in the oil content between two varieties at Stages S3 and S4. The protein content showed a trend of decreasing first, then increasing, reaching a minimum at Stage S4, and rebounding at Stage S5, which was consistent with an earlier study on changes in the protein content during the soybean seed development [19]. In the last two stages, the protein content of NMH was significantly lower than PXS.

### RNA-Seq analyses on developing seeds of two soybean landraces with contrasting seed hardness

**Figure 2:**
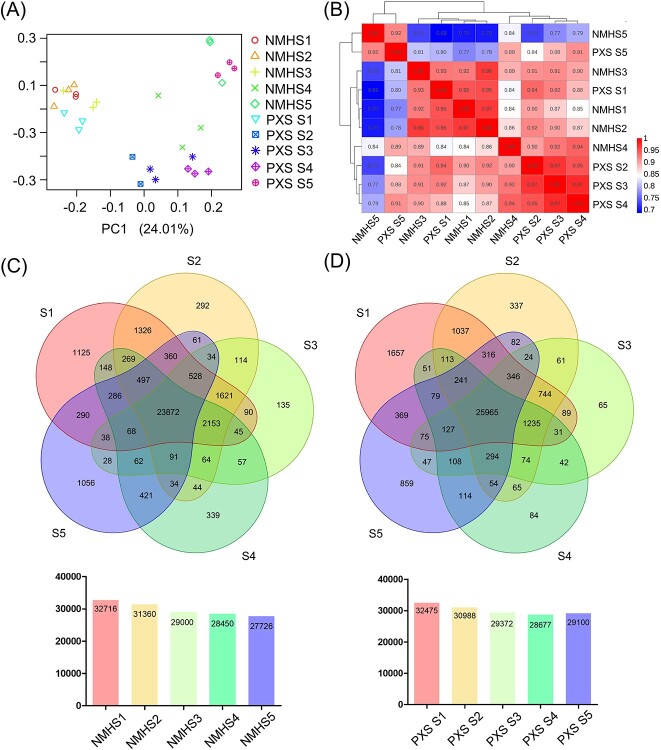
Global gene expression profiling during seed development. (**A**) PCA of the RNA-Seq data from five stages of seed development in NMH and PXS. (**B**) Spearman correlation coefficient analysis of RNA-seq data. (**C** and **D**) Venn diagrams of detected transcripts among the five stages of (**C**) NMH and (**D**) PXS.

In order to further investigate the molecular basis of the difference in seed development between the landraces, we performed RNA-Seq to generate transcriptome profiles. From Stages S1 to S5, soybean seeds from NMH and PXS were sampled with three biological replicates. A total of 30 libraries were constructed, and 247.34 Gb clean data were obtained. In different samples, the Q30 percentage was over 90.85%, and more than 94.03% of clean reads were mapped uniquely to the soybean genome (Table S2, see online supplementary material). In total, 32 716, 31 360, 29 000, 28 450, and 27 726 transcripts were identified in Stages S1, S2, S3, S4, and S5 of NMH, respectively. In addition, 23 872 transcripts were expressed at all five stages (Fig. 2C). Similarly, 32 475, 30 988, 29 372, 28 677, and 29 100 transcripts were identified in Stages S1, S2, S3, S4, and S5 of PXS, with 25 965 transcripts expressed at all stages (Fig. 2D). In general, with the development of seeds, the number of genes expressed at each stage gradually decreased. All expressed genes were divided into three expression levels based on FPKM [20]. In different samples, at least 60% of expressed genes showed low (0.5 ≤ FPKM ≤5) levels of expression. About 28.0% to 37.5% and 1.3% to 2.0% of the expressed genes exhibited moderate (5 ≤ FPKM ≤100) and high (FPKM ≥100) levels of expression, respectively (Fig. S2, see online supplementary material).

To investigate the massive quantity of information obtained from the transcriptome sequencing, principal component analysis (PCA) was conducted on all expressed genes in 30 samples (Fig. 2A). From the perspective of different stages, Stages S1, S2, and S3 of NMH were clustered together, suggesting that the overall transcriptome profiles were similar at these three developmental stages in NMH. Similarly, Stages S2, S3, and S4 of PXS were clustered together, indicating high similarity in their transcriptome profiles. From the perspective of different varieties, at Stages S1 and S5, high correlations were present between NMH and PXS. On the contrary, NMH and PXS showed obvious differences at Stages S2, S3, and S4, suggesting that S2, S3, and S4 could be the key stages leading to the phenotypic differences. Furthermore, we conducted hierarchical clustering and Spearman correlation coefficient analysis based on the average FPKM values for all the expressed genes in 30 tissue samples, as shown in Fig. 2B, which was almost consistent with the PCA results. Interestingly, the overall transcriptome profiles of the S2 and S3 samples were highly similar in both NMH and PXS, with correlation coefficients of 0.96 and 0.97, respectively. The S2 and S3 stages of NMH exhibited closer correlation with Stage S1, while the S2 and S3 stages of PXS tended towards the later (S4) stage of seed development. This observation indicated that PXS showed more rapid progression during the early and middle stages of seed development compared to NMH.

qRT-PCR analyses for seven genes from each developmental stage were performed to validate the quality of the RNA-seq data. The expression patterns of the tested genes obtained by qRT-PCR were highly similar to those observed in RNA-seq data (Fig. S3, see online supplementary material), indicating the consistency between the transcriptome and qRT-PCR analysis.

### Identification of differentially expressed genes during seed development

**Figure 3:**
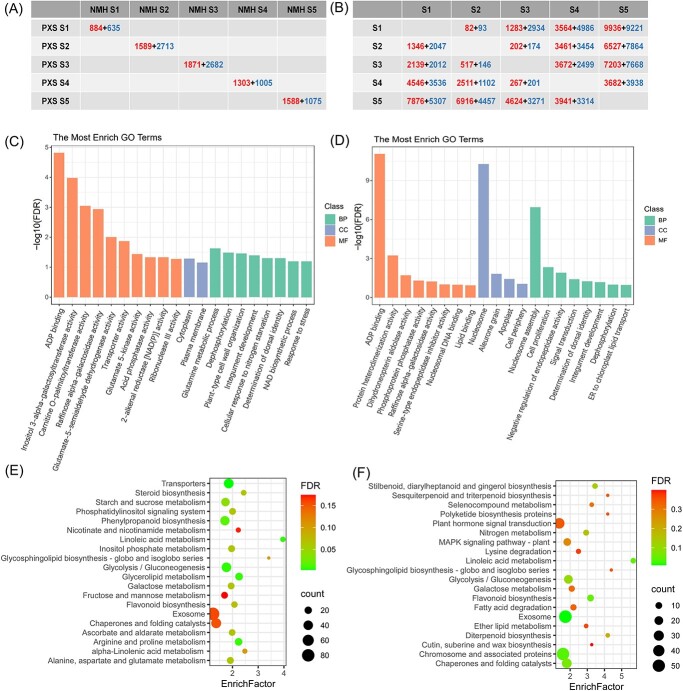
Differentially expression genes in different samples of PXS and NMH. (**A**) Numbers of DEGs between NMH and PXS at each developmental stage. (**B**) Numbers of DEGs in each developmental stage of NMH (upper-right portion) and PXS (lower-left portion). DEGs were filtered according to FPKM >5, FDR < 0.05, |log_2_ (fold change)| ≥ 1. Numbers in red or blue indicate the number of up-regulated or down-regulated genes when the sample corresponding to the row compared with the samples corresponding to the column, respectively. (**C**) and (**D**) The top 20 GO terms with the highest significance of S3 (**C**) and S4 (**D**). (**E**) and (**F**) The top 20 KEGG pathways with the highest significance of S3 (**E**) and S4 (**F**).

Pairwise comparisons were conducted to identify the differentially expressed genes (DEGs) between NMH and PXS at each developmental stage. Differential expression analysis showed that 1519 genes at the S1 stage, 4302 genes at the S2 stage, 4553 genes at the S3 stage, 2308 genes at the S4 stage, and 2663 genes at the S5 stage were found to be differentially expressed between NMH and PXS (|log_2_ (fold change)| ≥ 1 and FDR < 0.05, Fig. 3A). Pairwise comparisons were also conducted to identify the DEGs among the five developmental stages in each variety. The numbers of DEGs between different developmental stages were shown in Fig. 3B. Interestingly, there were 3393 genes significantly differentially expressed in pairwise comparison between Stages S1 and S2 of PXS, compared to 175 in NMH. On the contrary, there were 468 DEGs identified between Stages S3 and S4 of PXS, while 6171 DEGs were identified between Stages S3 and S4 of NMH. A large number of DEGs represent strong metabolic activity, accelerating the developmental progression of seeds. Compared with other adjacent stages, fewer DEGs were identified between Stages S2 and S3 in both NMH and PXS. These results were consistent with the information obtained from PCA and correlation analysis that the developmental progression of PXS was faster than that of NMH during the middle and early stages of seed development, and that the overall transcriptome profiles of the S2 and S3 stages were highly consistent. In view of the high consistency between the overall transcriptome profiles of the S2 and S3 stages, and considering that there was almost no significant phenotypic difference in seed hardness and seed component contents between the two varieties at Stage S2, we focused on Stages S3 and S4 in subsequent studies.

### Differential expression of genes determining seed development and seed hardness

To explore the function of the DEGs identified between NMH and PXS from Stages S3 and S4, which had been speculated to be the key stages determining seed hardness, GO enrichment analysis was performed and many GO terms were significantly enriched (FDR < 0.05). The top 20 GO terms with the highest significance identified from Stages S3 and S4 were shown in Fig. 3C and D, respectively. Notably, in the S3 stage, ‘glutamine metabolic process’ (GO:0006541), ‘integument development’ (GO:0080060), ‘plant-type cell wall organization’ (GO:0009664), and ‘transporter activity’ (GO:0005478) were significantly enriched. GO terms such as ‘nucleosome’ (GO:0000786), ‘nucleosome assembly’ (GO:0006334), ‘nucleosomal DNA binding’ (GO:0031492), and ‘cell proliferation’ (GO:0010456) were significantly enriched at Stage S4, and most of the genes involved were down-regulated in PXS (Fig. S4, see online supplementary material), indicating that cell mitosis and DNA replication were still quite active in Stage S4 of NMH, which may slow down the accumulation of stored substances such as oil and storage protein in the seeds, resulting in lower seed hardness.

KEGG pathway enrichment analysis was also conducted to identify significantly enriched metabolic and signal transduction pathways in DEGs identified from Stages S3 and S4. The top 20 KEGG pathways with the highest significance identified from Stages S3 and S4 are shown in Fig. 3E and F. ‘Transporters’ (KO02000), ‘Glycerolipid metabolism’ (KO00561), ‘Linoleic acid metabolism’ (KO00591), ‘Glycolysis / Gluconeogenesis’ (KO00010), ‘Arginine and proline metabolism’ (KO00330), ‘Phenylpropanoid biosynthesis’ (KO00940), ‘Starch and sucrose metabolism’ (KO00500), and ‘Alanine, aspartate and glutamate metabolism’ (KO00250) were significantly enriched at Stage S3. ‘Exosome’ (KO04147), ‘Linoleic acid metabolism’ (KO00591), ‘Chromosome and associated proteins’ (KO03036), ‘Glycolysis / Gluconeogenesis’ (KO00010), ‘Nitrogen metabolism’ (KO00910), ‘Fatty acid degradation’ (KO00071), and ‘Lysine degradation’ (KO00310) were enriched at Stage S4.

During Stages S3 or S4, some synthetic and metabolic pathways that may affect seed hardness are significantly enriched. The expression patterns of key genes involved in starch synthesis, starch degradation, fatty acid synthesis, storage protein synthesis, lignin synthesis, and DNA replication were analysed (Fig. S4, see online supplementary material). The expression levels of most genes encoding starch synthase were found to be lower in PXS compared with NMH during Stage S4. Most genes involved in starch degradation were upregulated in PXS during Stages S3 and S4 (and especially at Stage S3), including genes encoding α-amylase, β-amylase, and phosphorylase. Compared with NMH, the genes encoding seed storage proteins exhibited higher transcriptional activity in PXS during Stages S4 and S5. In particular, two genes encoding beta conglycinin, *Glyma.20G146200* and *Glyma.20G148200*, were upregulated in PXS from S1 to S5, with more than six-fold upregulation. At Stages S4 and S5, most of the genes involved in fatty acid synthesis and elongation were upregulated in PXS, encoding proteins such as pyruvate dehydrogenase, acetyl-CoA carboxylase, and acetyl carrier protein. Higher expression of most of the genes which encode proteins related to phenylpropane metabolism and lignin synthesis, such as CAD, 4CL, HCT, and CCoAOMT could be observed in PXS, particularly at Stages S3 and S5. At Stages S2 and S3, the transcription levels of most genes involved in DNA replication and cell proliferation were higher in PXS compared to NMH, but the opposite was true in Stage S4.

### Weighted gene co-expression network construction and hub gene determination

**Figure 4:**
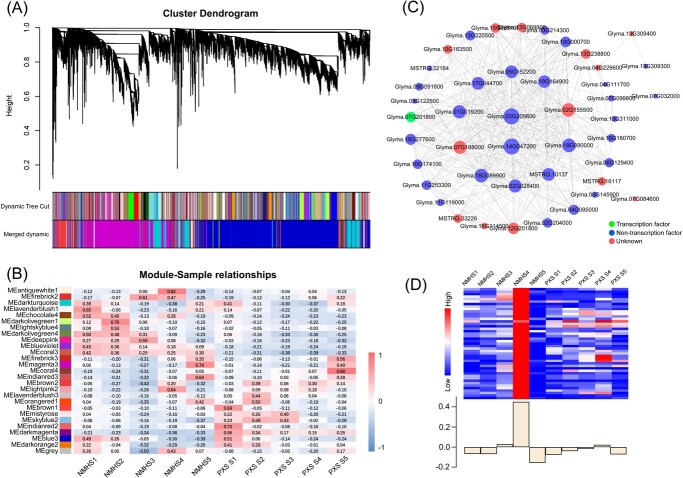
WGCNA of expressed genes in NMH and PXS. (**A**) Cluster dendrogram showing co-expression modules identified by WGCNA. Each leaf (short vertical line) in the tree represents one gene. The branches correspond to modules of highly interconnected genes. The color rows below the dendrograms indicate the division of the modules according to the clustering results and 27 merged modules based on hierarchical clustering. (**B**) Module-sample relationships based on Pearson correlation coefficients. Each row corresponds to a module indicated by different colors. Each column corresponds to a sample from different stages of NMH or PXS. (**C**) The gene co-expression network map of the antiquewhite1 module. Node size denotes gene connectivity, and node color represents gene category. The candidate hub genes are located at the center of each module. (**D**) Heatmap shows the expression pattern of all the co-expressed genes in antiquewhite1 module. The histogram shows the variation in module eigengenes expressed in different samples.

The gene regulatory network during seed development was investigated using WGCNA. After screening out the genes with a low expression (FPKM < 0.5), 31 459 genes were retained for the WGCNA. Based on the co-expression patterns of these genes, the gene cluster dendrogram was constructed and 27 distinct modules (labelled with different colors) were identified (Fig. 4A). Next, the expression patterns of each module in different samples were analysed based on the module eigenvalues (Fig. 4B). Seven modules showed opposite expression patterns between the two varieties at Stage S3 or S4 (Fig. 4D; Fig. S5A, see online supplementary material). Genes in the antiquewhite1, darkolivegreen4, firebrick2, and deeppink modules were highly expressed in NMH, but exhibited low expression levels in PXS at Stages S3 or S4. Genes in the mistyrose and skyblue2 modules were highly expressed in PXS, but were lowly expressed in NMH at Stages S3. In addition, the coral3 module showed variety specificity and was highly expressed in only one variety. We constructed gene co-expression network maps of these seven modules (Fig. 4C;Fig. S5B–G, see online supplementary material), where the genes at the center of each network were the hub genes of the module. All hub genes belonging to each module are listed in Table S3 (see online supplementary material).

To clarify the role of the seven modules in the soybean seed development, we conducted KEGG enrichment analyses; the top 10 KEGG pathways with the highest significance identified from each module are shown in Fig. S6 (see online supplementary material). Through KEGG enrichment analysis, we found that these seven modules may play roles in different aspects, jointly regulating the main storage substance contents and seed hardness of soybeans. The deeppink, mistyrose, darkolivegreen4, and coral3 modules showed enrichment in the KEGG pathway related to protein processing, such as ‘Protein processing in endoplasmic reticulum’ (KO04141) and ‘Protein processing’ (KO99975). The firebrick2 module was enriched in ‘Fatty acid elongation’ (KO00062), ‘Lipid biosynthesis proteins’ (KO01004), and ‘Pyruvate metabolism’ (KO00620). Therefore, we speculate that this module was mainly involved in regulating the oil content of soybean seeds. Skyblue2 module was involved in starch and sucrose metabolism and cell wall synthesis. ‘Starch and sucrose metabolism’ (KO00500) and ‘Phenylpropanoid biosynthesis’ (KO00940) were significantly enriched in this module. Genes encoding cell wall synthesis-related proteins such as caffeic acid 3-O-methyltransferase (COMT), cellulose synthase A catalytic subunit 2 (CesA2), and shikimate O-hydroxycinnamoyl transferase (HCT) were identified in this module. Notably, antiquewhite1 module was widely involved in the synthesis and accumulation of starch, protein, and fatty acids at Stage S4 of soybean development. The antiquewhite1 module includes 45 genes, in which ‘Pyruvate metabolism’ (KO00620), ‘Glycolysis/Gluconeogenesis’ (KO00010), ‘Carbohydrate metabolism’ (KO09101), ‘Starch and sucrose metabolism’ (KO00500), ‘Phenylalanine, tyrosine and tryptophan biosynthesis’ (KO00400), and ‘Citrate cycle (TCA cycle)’ (KO00020) were enriched. A sugar will eventually be exported transporter (SWEET) encoding gene (*Glyma.03G209600*) and a nitrate transporter 1/peptide transporter family (NPF) gene (*Glyma.14G047200*) were identified as candidate hub genes for this module. In addition, genes encoding soluble starch synthase (*Glyma.06G129400*), pyruvate kinase (*Glyma.19G000700*), and pectinesterase (*Glyma.17G044700*) were also detected in this module.

### *GmSWEET2* increased soybean seed hardness at Stage R6

**Figure 5:**
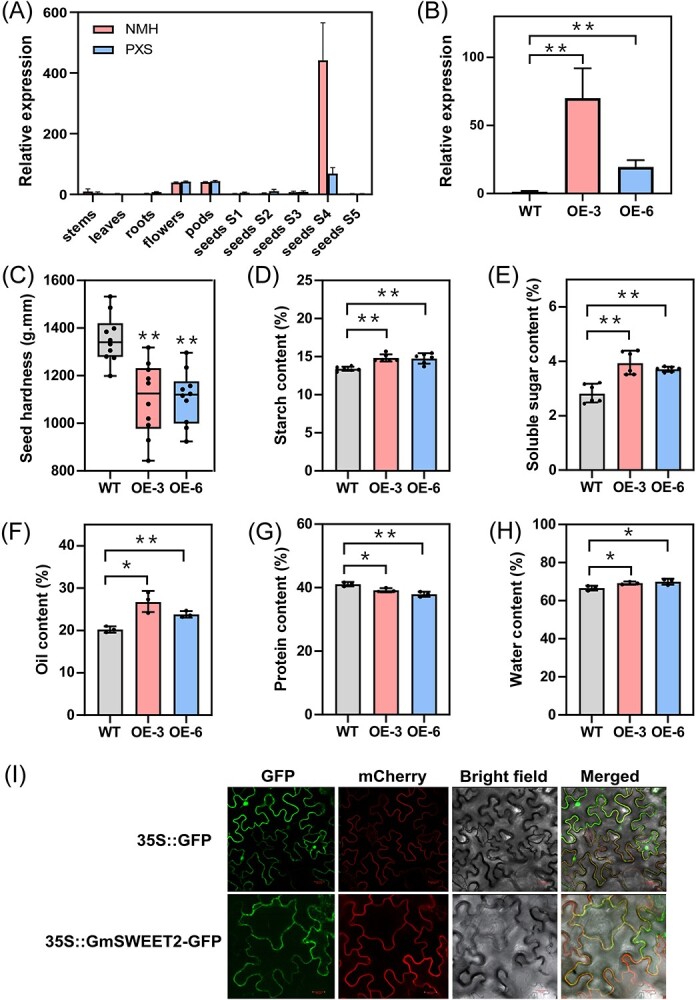
*GmSWEET2* alters the seed hardness and component contents at Stage R6. (**A**) Tissue-specific expression of *GmSWEET2.* The relative expression of *GmSWEET2* in soybean stems, leaves, roots, flowers, pods, and seeds at different developmental stages was determined by qRT-PCR. (**B**) The relative expression level of *GmSWEET2* in WT (PXS) and *GmSWEET2*-OE lines. Data are presented as mean ± SD (*n* = 3 biological replicates). ^*^P < 0.05, ^**^P < 0.01 compared with WT, Student’s *t*-test. (**C**) Seed hardness of WT (PXS) and *GmSWEET2*-OE lines at Stage R6. The box plot shows median (horizontal line) and individual values (black dots) (*n* = 8 biological replicates). ^*^P < 0.05, ^**^P < 0.01 compared with WT, Student’s *t*-test. (**D**–**H**) Starch, soluble sugar, oil, protein, and water contents in WT (PXS) and *GmSWEET2*-OE lines at Stage R6; data are presented as mean ± SD [*n* = 6 for (**D**) and (**E**), *n* = 3 for (**F**) to (**H**)]. ns, no significant difference. ^*^P < 0.05, ^**^P < 0.01, Student’s *t*-test. (**I**) The fluorescence imaging of 35S::GmSWEET2-GFP expressed fusion protein in cells of *Nicotiana benthamiana* leaves. Bars = 20 μm.

A sugar will eventually be exported transporter (SWEET) gene *Glyma.03G209600* was identified as a hub gene of the antiquewhite1 module. In a previous study on the soybean SWEET gene family, *Glyma.03G209600* was designated as *GmSWEET2* [21], which is a putative ortholog of *AtSWEET9* and transport sucrose across the plasma membrane. We compared the coding DNA sequence (CDS) of *GmSWEET2* from NMH and PXS. As shown in Fig. S7A (see online supplementary material), the CDS of *GmSWEET2* in NMH and PXS are consistent. Therefore, we speculate that the difference in seed hardness between NMH and PXS may be caused by difference in the expression levels of *GmSWEET2*. The tissue expression pattern of *GmSWEET2* was verified by qRT-PCR (Fig. 5A). *GmSWEET2* was almost exclusively expressed in sink organs such as flowers, pods, and S4-stage seeds, indicating that GmSWEET2 play an important role in the unloading of sucrose in sink organs, especially in the transport of sucrose from the maternal tissue to the seeds during the later stages of seed development. In Stage S4, compared to PXS, it showed a higher expression level in the seeds of NMH.

To assess the role of *GmSWEET2* in the determination of soybean seed hardness, we generated a series of *GmSWEET2*-overexpressing lines (*GmSWEET2*-OE) in Pixiansilicao (PXS) genetic background (Fig. 5B). There was no significant difference in seed size between transgenic plants and wild types at Stage R6 (Fig. S9, see online supplementary material). However, *GmSWEET2* overexpression led to a significant decrease in seed hardness at Stage R6 compared with wild type (WT) plants (Fig. 5C). To further understand the mechanism of *GmSWEET2*-induced changes in the hardness of soybean seeds, we measured the main seed component contents of wild type and *GmSWEET2*-OE lines at Stage R6, including starch, soluble sugar, oil, protein, and water contents (Fig. 5D–H). Compared with the wild type, the starch, soluble sugar, oil, and water contents were significantly increased in the *GmSWEET2*-OE plants, while the protein content was significantly decreased, which endows the over-expressed plants with a lower seed hardness at Stage R6. To reveal the subcellular localization of GmSWEET2 protein, *GmSWEET2* was expressed in fusion with GFP in tobacco leaves. As shown in Fig. 5I, the GmSWEET2-GFP fusion protein was localized in plasma membrane.

## Discussion

### The content of protein and starch is the key factor determining the hardness of vegetable soybean

Seed hardness is one of the main indicators of vegetable soybean edible quality [22]. Hard texture generally decreases consumer acceptance of the vegetable soybean. Therefore, understanding the molecular mechanisms controlling seed hardness is important for improving the edible quality of vegetable soybean. In this study, two soybean landraces, NMH (with stable low seed hardness) and PXS (with stable high seed hardness), were screened from 216 soybean accessions. The hardness difference between NMH and PXS was significant at Stage S3, which represented the late of R5 (beginning seed) stage, and reached the maximum at Stage S4 (this corresponds to the R6 (full seed) stage of soybean reproductive physiological stages, which is also the harvest period of vegetable soybean [23]). As a physical property of seeds, hardness is largely affected by the chemical composition of the seeds. Soluble sugar such as sucrose, glucose, and fructose contribute to the sweetness of vegetable soybean [24]. Previous studies on sensory evaluations of different vegetable soybean varieties have shown a negative correlation between chewiness and sweetness of vegetable soybeans [3]. However, no significant difference in the soluble sugar content was observed in our experiments between the two varieties at Stage S4. Seed hardness was also found to be positively correlated with the protein content and negatively correlated with the starch content in studies on rice, maize, and chickpea [25–27]. In Stage S4, PXS had higher protein content, and lower starch content compared to NMH. Previous studies on wheat have found that the hardness of wheat grains seems to be determined by the physical structure of the endosperm protein matrix and starch granules. Hard wheat has sufficient protein to form a continuous protein matrix to entrap starch grains physically, while soft wheat cannot form a continuous protein matrix to wrap starch granules, resulting in a softer and more open-grained structure [28]. The observation on the microstructure of vegetable soybean seeds at Stage R6 also found that the high-hardness variety had a tighter protein meshwork structure, while seeds of the low-hardness variety had a lower protein content and a looser protein structure [17]. The decrease in the protein : starch ratio leads to a more open seed structure, with more water filling the gaps between starch grains and the protein matrix, further reducing seed hardness. We also observed that the oil content in high seed hardness landrace PXS was significantly higher than that in low seed hardness landrace NMH at Stage S3 and S4. However, some studies have shown a significant negative correlation between seed hardness and oil content in mature soybeans, and studies with vegetable soybeans have shown no significant correlation between oil content and seed hardness [9, 17]. The relationship between the oil content and the hardness of vegetable soybean seeds still needs further study to prove.

### Transcriptome analysis revealed the key pathway involved in the regulation of vegetable soybean seed hardness

Similar to the phenotype of seed hardness, the protein, starch, oil, and water contents between NMH and PXS mostly showed significant differences in Stages S3 and S4. Therefore, we speculate Stages S3 and S4 are the key stages determining the hardness of vegetable soybean seeds. PCA and DEG analysis showed that the transcriptome profiles of PXS in Stage S1 and NMH in Stages S1, S2, and S3 were relatively similar, while PXS in Stages S2, S3, and S4 and NMH in Stage S4 had similar gene expression profiles, suggesting that the seed development progression of NMH was slower than PXS at middle and early stages of seed development. Studies on chickpea and maize have shown that early rapid development may be related to smaller grain sizes, whereas in soybean, seed size tends to be negatively correlated with seed hardness [6, 29, 30].

KEGG pathway analysis showed that the DEGs of NMH and PXS in Stage S3 were significantly enriched in the pathways related to amino acid metabolism, such as ‘arginine and proline metabolism’ and ‘alanine, aspartate and glutamate metabolism’. Amino acid is the basic element of protein. Interestingly, the metabolites of these pathways, such as asparagine, glutamine, and arginine, are the highest concentrations of amino acids during the peak protein-accumulation stages (Stages R5 and R6) [31]. As important sources of nitrogen for filling soybeans, the contents of asparagine and glutamine are positively correlated with the seed protein content [32]. In addition, ‘Glycolysis/Gluconeogenesis’ was significantly enriched in the DEGs of both Stages S3 and S4, and the levels of expression of genes contained in this pathway were higher in NMH than in PXS. Previous studies have found that the prolonging of mitotic activity in maize and chickpea was accompanied by higher gluconeogenesis [29, 30]. Gluconeogenesis does not occur when seeds receive adequate supplies of sugars [33]; however, in the late stage of soybean seed development, as the maternal nutritional supply decreases, pyruvic acid and glycerol are used as raw materials through the gluconeogenesis step to continuously accumulate carbohydrates [31]. Therefore, we observed that the rate of accumulation of oil in seeds decreased significantly from Stage S4 to Stage S5 due to the reduction of maternal nutritional supply and the consumption of gluconeogenesis. However, there was no increase in starch and soluble sugar contents, which could be attributed to the degradation of starch in the late stage of seed development into soluble sugar, which is then converted into fatty acids, glycerol phosphate, and amino acids, as has been confirmed in other species [34, 35]. ‘Phenylpropanoid biosynthesis’ was significantly enriched in DEGs at Stage S3, and the expression levels of most genes encoding key enzymes of phenylpropane metabolism and lignin synthesis such as 4CL, C4H, and HCT were higher in PXS. The phenylpropane metabolic pathway is one of the important secondary metabolic pathways in plants, and lignin is an important metabolite of phenylpropane metabolism. Early studies have shown that lignin can impart higher seed coat strength to legume crops such as soybeans and chickpeas [13, 14, 36]. ‘Starch and sucrose metabolism’ were significantly enriched at Stage S3. Additionally, compared with NMH, we found that most genes involved in starch degradation exhibited higher transcriptional activity in PXS at Stage S3, while genes encoding starch synthase mostly exhibited lower transcriptional activity in PXS at Stage S4. This observation explains why the starch content of PXS was higher than that of NMH in Stages S3 and S4. Genes involved in fatty acid synthesis and elongation, as well as genes encoding storage proteins, exhibited higher transcriptional activity in PXS at Stages S4 and S5, which was consistent with the observed higher protein and oil contents in PXS at Stages S4 and S5.

### *GmSWEET2* plays an important role in regulating the hardness of vegetable soybean seeds

As a powerful tool in systems biology, WGCNA has been used to identify key genetic networks involved in many crops. In this study, we found that there were seven modules displayed opposite expression patterns between NMH and PXS at Stages S3 or S4. Through KEGG pathway enrichment analysis of these seven modules, we identified that the antiquewhite1 module is mainly related to the metabolism and accumulation of components such as protein, starch, and fatty acids.

The antiquewhite1 module was highly expressed in Stage S4 of NMH. A sugar will eventually be exported transporter encoding gene *GmSWEET2* and a nitrate transporter1/peptide transporter family (NPF) protein encoding gene *GmNPF6.4* were identified as the hub genes of this module. Sucrose is the main source of carbon energy transferred to developing seeds through the phloem [37]. It relies on sucrose carriers and sucrose efflux transporters to achieve effective transportation and distribution [38, 39]. SWEETs are considered to play important roles in sugar translocation to seeds and consequently affect seed development and chemical composition [40, 41]. *GmSWEET2* was located in a reported seed protein content QTL [42]. NPF transporters are widely involved in the process of nitrogen absorption and utilization by plants, and play important roles in improving the nitrogen-utilization efficiency and yield of crops. Previous studies on rice and maize have shown that NPF transporters affect grain development and the contents of storage substances such as starch and protein [43–45].

In this study, overexpression of *GmSWEET2* resulted in an increase in the starch, soluble sugar, and oil contents, and a decrease in the protein content in the seeds at Stage R6. GmSWEET2 was localized in plasma membrane and was highly expressed in R6-stage seed, mediating the unloading of sucrose to developing seeds. In the storage organs of plants, the raw material for starch synthesis mainly comes from the sucrose synthesized in the leaves, which is transported over long distances to the storage organs through the phloem [46]. Meanwhile, sucrose is also the primary source of acetyl CoA, the precursor for lipid biosynthesis [47]. When the level of expression of *GmSWEET2* increases, it will be accompanied by more sucrose transfer from the maternal tissue to the developing seeds, resulting in more starch and oil accumulation in seeds. In addition, protein synthesis depends on the availability of carbon and nitrogen. As the activity of SWEET sugar transporters increases, the availability of nitrogen may become a limiting factor, and more intermediates of glycolysis diverted to the fatty acid synthesis pathway, thereby reducing relative protein content [40]. The decrease in protein : starch ratio leads to the inability of seeds to form a tighter protein meshwork structure to wrap starch granules, resulting in a softer and more open seed structure, thereby reducing seed hardness. In this study, we also used CRISPR/Cas9 to knock out the *GmSWEET2* genes in Niumaohuang (NMH), and obtained two homozygous *GmSWEET2*-knockout lines (*GmSWEET2*-KO, Fig. S8A and B, see online supplementary material). However, there was no significant difference in seed hardness at Stage R6 between *GmSWEET2*-KO plants and wild type plants (Fig. S10, see online supplementary material). This may be because the SWEET family contains many genes that work together and have certain redundancy, resulting in other genes of the SWEET family replacing the role of *GmSWEET2* to a certain extent after *GmSWEET2* was knocked out. Although the expression level of *GmSWEET2* exhibited a significant difference between NMH and PXS, the promoter sequence of *GmSWEET2* was consistent in the two landraces (Fig. S7B, see online supplementary material). This may be due to differences in the transcription factors that bind to the promoter and regulate *GmSWEET2* expression between NMH and PXS. According to the prediction of PlantPAN3.0 [48], 905 transcription factors have the potential to bind with *GmSWEET2* promoter, of which 35 transcription factors are differentially expressed in PXS and NMH, including transcription factor families such as ERF, NAC, WRKY, and bHLH (Table S5, see online supplementary material). The specific role of these transcription factors in regulating the hardness of vegetable soybean seeds require further study.

To summarize, GmSWEET2 sugar transporter protein can reduce the hardness of R6-stage soybean seeds by increasing the starch content and reducing the protein content. The analyses of the comprehensive transcriptome data set in this study provide a useful genomic resource for research on the quality traits of vegetable soybean, and new insights into the molecular network of soybean seed development.

## Materials and methods

### Plant materials and sampling

In 2014, 2015, and 2016, 216 soybean accessions from 26 provinces in China were planted under natural conditions at Jiangpu Agricultural Research Farm (32°12′N, 118°37′E), Nanjing, China. The cultivation followed a randomized complete block design with three replications. Ten pods were picked off at Stage R6 to test seed hardness. Two soybean landraces with stable difference in seed hardness in multiple environments were selected for further analysis. The low seed hardness landrace Niumaohuang (NMH, Chinese Crop Germplasm Information System, accession number ZDD00610), and the high seed hardness landrace Pixiansilicao (PXS, Chinese Crop Germplasm Information System, accession number ZDD03741) were grown in the field in 2018. The two groups of soybean seed samples from NMH and PXS were collected in three biological replicates at 20, 25, 30, 35, and 40 days after flowering (DAF), representing Stages S1, S2, S3, S4, and S5, respectively. Thus, 30 samples were collected. The tissue samples were snap-frozen in liquid nitrogen and stored at −80°C.

### Illumina sequencing and identification of DEGs

Extracted total RNA from each sample, tested the purity, concentration, and integrity, and send 0.5-2 μg RNA for library construction and RNA-seq (Genepioneer Biotechnologies, Nanjing, China). The Illumina high-throughput sequencing platform (HiSeq/MiSeq) was used for cDNA library sequencing. The raw data generated by sequencing was preprocessed for quality using Trimmomatic software [49]. HISAT2 was used to map the clean reads to the reference genome of soybean (*G. max* Wm82. a4. v1) [50]. The FPKM value of transcript or gene expression was calculated using Cufflinks [51]. ‘DEseq2’ R package was used for the identification of DEGs which were filtered with|log_2_ (fold change)| ≥ 1 and false discovery rate (FDR) < 0.05.

### GO and KEGG enrichment analysis

Functional annotation of all DEGs was performed based on the Gene Ontology (GO) database and the Kyoto Encyclopedia of Genes and Genomes (KEGG) database. Significantly enriched terms or pathways were identified using TBtools with a threshold of FDR < 0.05 [58].

### Seed hardness and chemical composition contents

Texture analyser (Stable Micro System TA. XT *Plus*) was used to test the hardness of immature seeds according to the method described by our previous report [18]. Briefly, a Φ2 mm probe was equipped for puncture testing at a testing speed of 1 mm/s, with a testing depth set to 2 mm. The mechanical work (W) measured during the puncture process of the texture analyser probe was used to evaluate the hardness of the seeds. After measuring the seed hardness, all samples were blanched in a drying oven at 105°C for 30 minutes, then the samples dried to a constant weight at 65°C. The water content was measured by the gravimetric analysis method. The contents of starch and soluble sugar were measured by the anthrone colorimetry method [52], the content of protein was measured by the Kjeldahl digestion method [53], and the content of oil was measured by the acid hydrolysis method.

### Weighted gene co-expression network analysis and hub gene finding

After screening out undetectable or low expression genes, genes with FPKM of more than 0.5 were utilized for WGCNA using the ‘WGCNA’ R package [54]. The power value was set to 11 to make the gene co-expression network conform to a scale-free network. The ‘mergeCutHeight’ was set to 0.25, and the ‘minModuleSize’ was set to 25 to further classify and merge the gene modules. The module eigengene, *E*, was calculated to identify the modules related to seed hardness. In addition, for each module, the top one-third pairs of genes with the highest weighted value were selected to visualize the co-expression network using Cytoscape (v.3.8.0).

### Quantitative real-time PCR validation

To validate the RNA-seq results, qRT-PCR was used to determine the relative expression levels of seven DEGs in each variety and stage. Primer Premier 5 software was used to design the gene-specific primers, which were listed in Table S4 (see online supplementary material). Soybean gene *GmActin11* (*Glyma.18G290800*) is the internal control gene and fold change was calculated using the 2^-ΔΔCT^ method [55]. Three independent biological replicates were performed on each sample to ensure statistical reliability.

### Construction of *GmSWEET2* overexpression vectors and plant transformation

We cloned the full-length coding sequence (CDS) of *GmSWEET2* into the *pTF101.1* vector for overexpression of *GmSWEET2* under the control of the CaMV 35S promoter (*35S::GmSWEET2*), and then the recombinant *35S::GmSWEET2* plasmid vector was transformed into the high seed hardness soybean variety Pixiansilicao using *Agrobacterium tumefaciens* strain EHA101. Plant transformation was done according to a previously reported protocol [56]. Positive plants were validated by amplifying the 35S promoter and selecting marker genes (*bar*) using the PCR technique, and Basta screening was also used to verify positive plants. The expression level of *GmSWEET2* in overexpressed plants was verified through qRT-PCR analysis (Fig. 5B). T_3_ homozygous transgenic lines were used for phenotypic evaluation. Primers used are listed in Table S4 (see online supplementary material).

### Construction of *GmSWEET2* CRISPR/Cas9 vectors and plant transformation

Low seed hardness soybean variety Niumaohuang was used for tissue culture and transformation. Using the CRISPR-P2.0 website, four target sites were designed based on the exons of the GmSWEET2 gene. CRISPR/Cas9 vector, pGES201, was used for genome editing. The vector was constructed according to the previously reported protocol and transformed into *A. tumefaciens* strain EHA105 via electroporation [57]. Plant transformation was performed according to a previously reported protocol [56].

Genomic DNA isolated from each individual plant in the T_0_ generation was used to identify the gene editing plants by PCR and DNA sequencing. Primers used are listed in Table S4 (see online supplementary material). Heterozygous mutations showed overlapping peaks in the target sites; however, there was no overlapping peak observed in wild type (WT) and homozygous mutations. Homozygous mutations were identified by sequence alignment with the wild type sequence and were used for further analysis (Fig. S5B and C, see online supplementary material).

### Subcellular localization

Coding sequences of *GmSWEET2* was introduced into *pBin-GFP4*, and placed under the control of the CaMV 35S promoter. The recombinant plasmids and empty vectors were transiently transformed into *Nicotiana benthamiana* leaves using *A. tumefaciens* strain GV3101. After 2–3 days, the fluorescence signals were observed using a confocal laser scanning microscope (CLSM, Leica SP8, Germany). The excitation wavelengths were 488 nm and 580 nm for green fluorescent protein (GFP) and mCherry, respectively.

## Supplementary Material

Web_Material_uhae084

## Data Availability

All data are available in the article and in the online supplementary data.
