# Supplementary material for: Genome-wide transcriptome analysis reveals key regulatory networks and genes involved in the determination of seed hardness in vegetable soybean
Source: Hortic Res. 2024 Apr 2;11(5):uhae084. doi: 10.1093/hr/uhae084 (PMC11101316; doi:10.1093/hr/uhae084)
Supplement: Web_Material_uhae084 [file web_material_uhae084.zip › Supplementary figure.docx]

# Genome-wide transcriptome analysis reveals key regulatory networks and genes involved in the determination of seed hardness in vegetable soybean

**Congcong Wang, Jianyu Lin, Yuanpeng Bu, Ruidong Sun, Yang Lu, JunYi Gai, Han Xing**^*^**, Na Guo**^*^**, Jinming Zhao**^*^

Key Laboratory of Biology and Genetics Improvement of Soybean, Ministry of Agriculture / Zhongshan Biological Breeding Laboratory (ZSBBL) / National Innovation Platform for Soybean Breeding and Industry-Education Integration / State Key Laboratory of Crop Genetics & Germplasm Enhancement and Utilization / College of Agriculture, Nanjing Agricultural University, Nanjing 210095, China


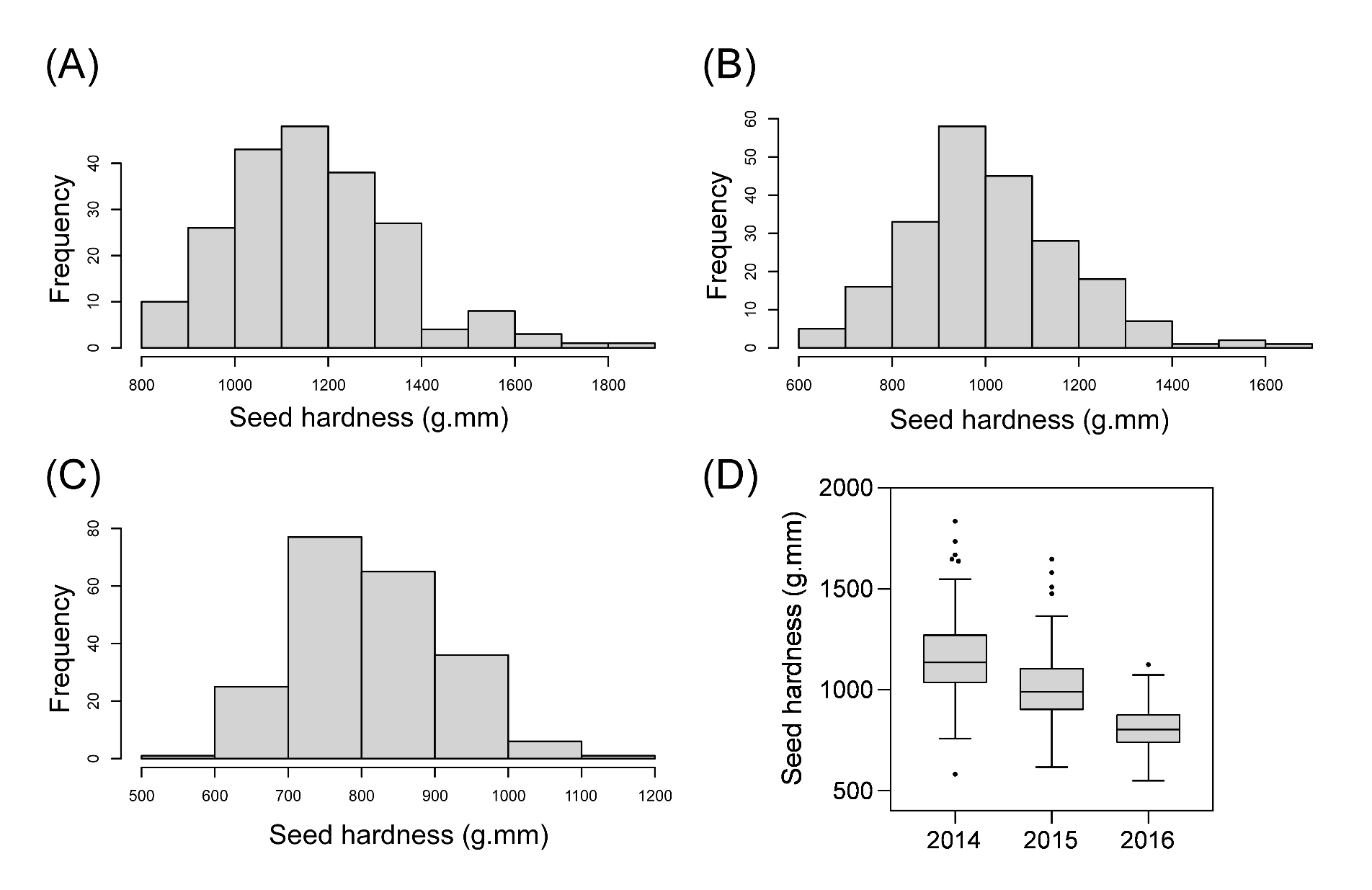


Fig. S1

Phenotypic distribution of seed hardness at the R6 stage in the 216 soybean accessions over three years. (A-C) The frequency distribution of seed hardness at the R6 stage in the 216 soybean accessions for 2014, 2015, and 2016. (D) Boxplots of seed hardness over 3 years.


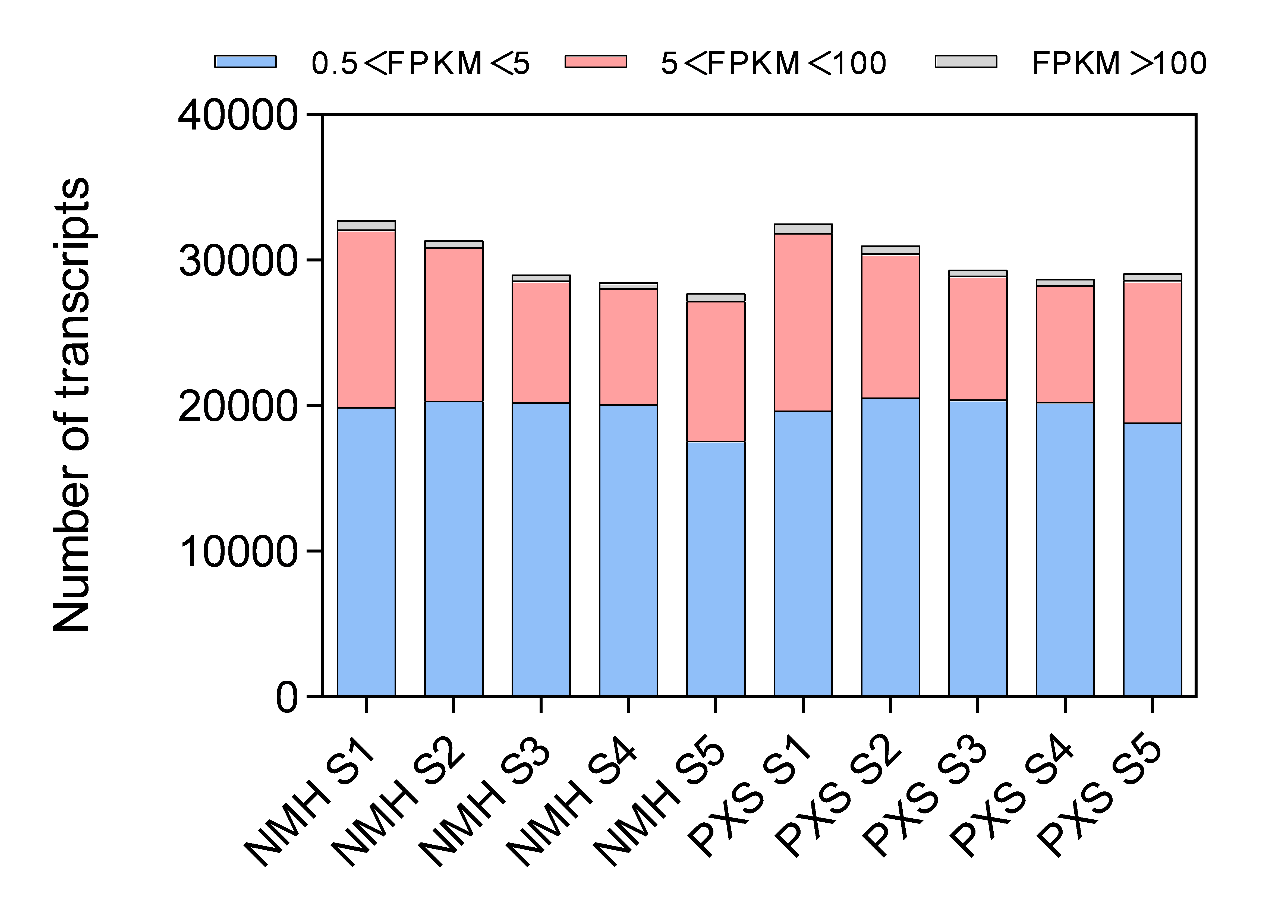


Fig. S2

Numbers of detected transcripts in each sample.


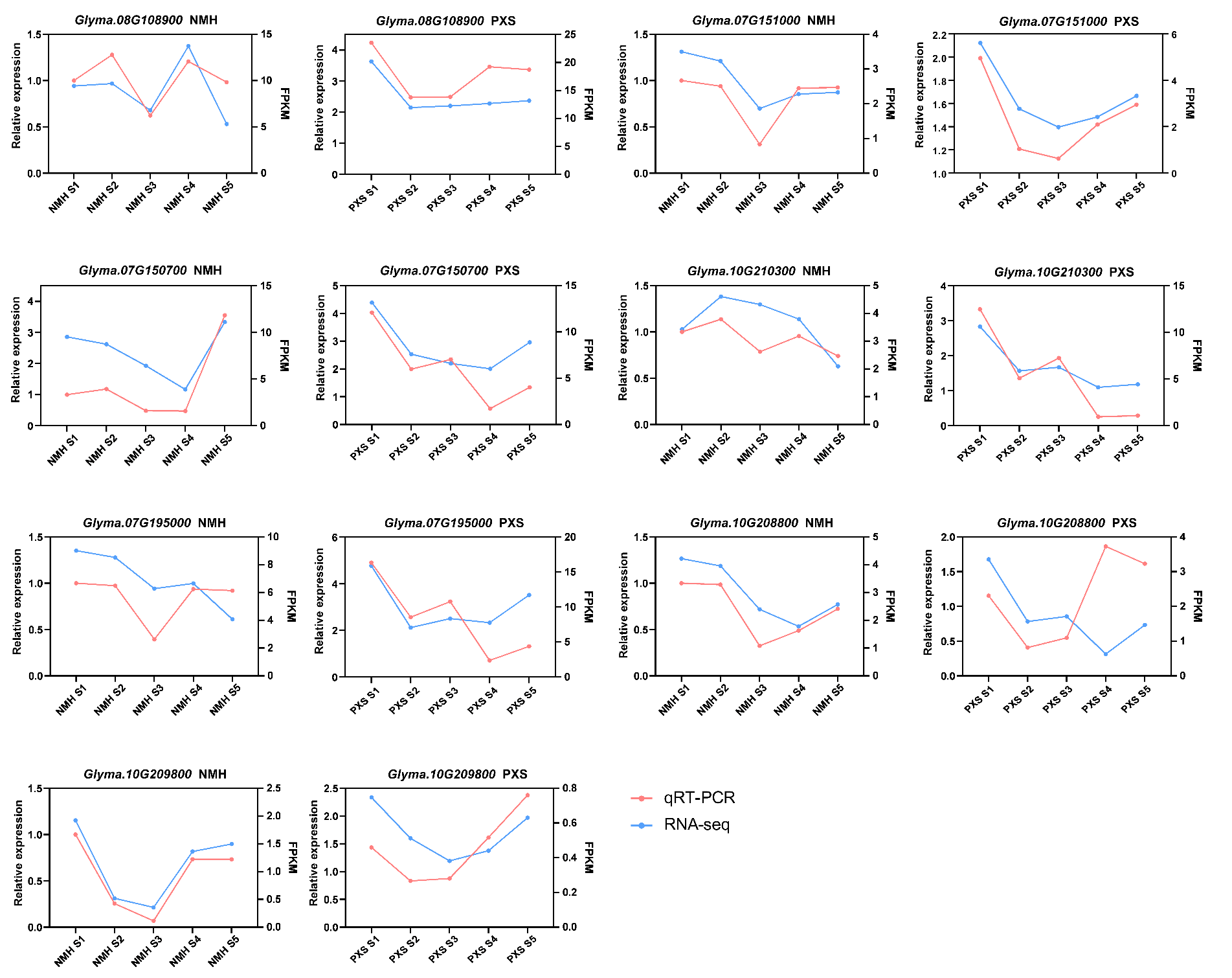


Fig. S3

qRT-PCR verification of seven selected genes.


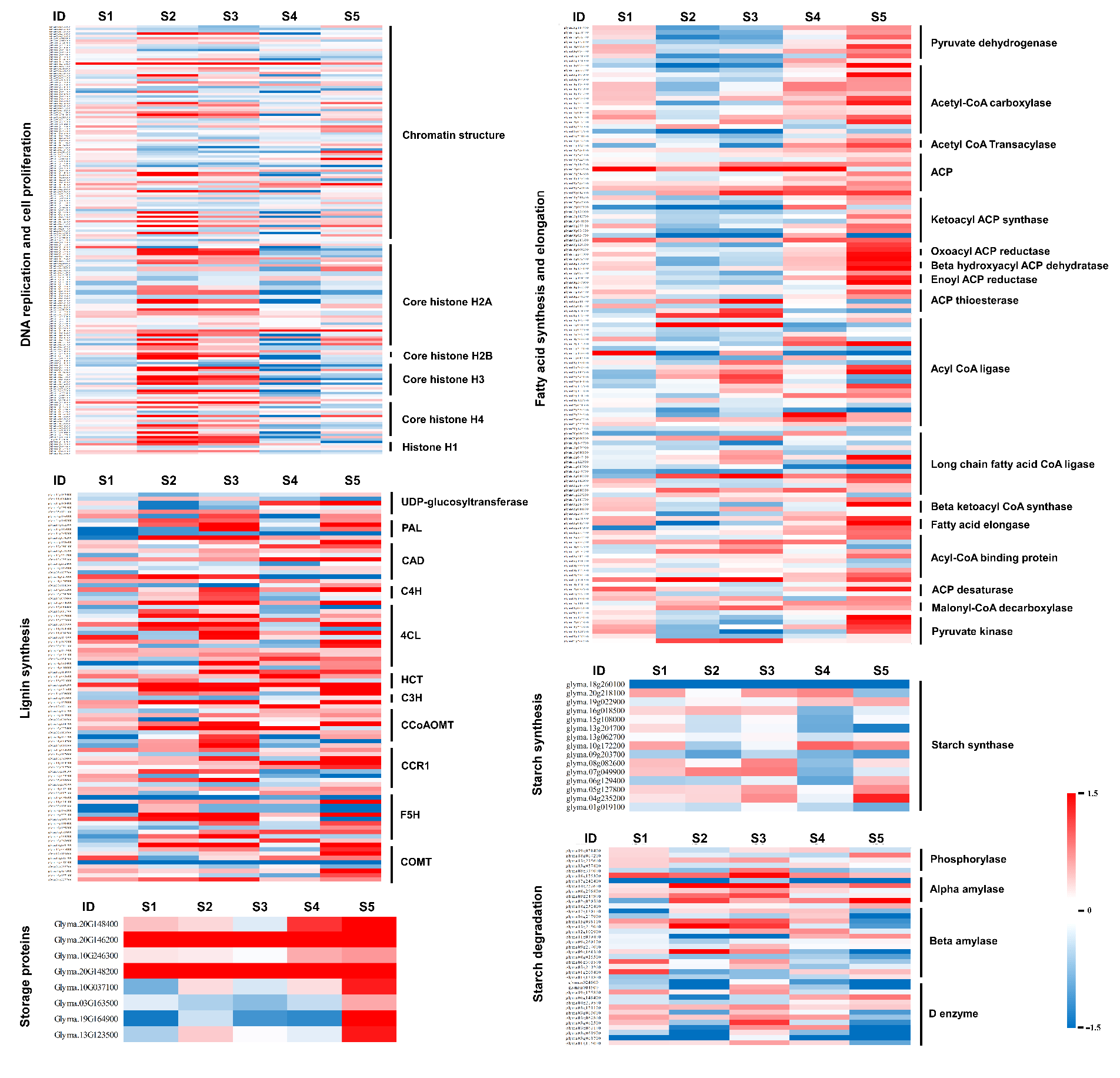


Fig. S4

Differential expression of genes performing specific functions in NMH and PXS. The expression pattern of each gene is shown by five cells, which represent the relative log2 (fold change) at Stages S1-S5, respectively. Red and blue colors denote higher and lower expressions, respectively, in PXS as compared with NMH.


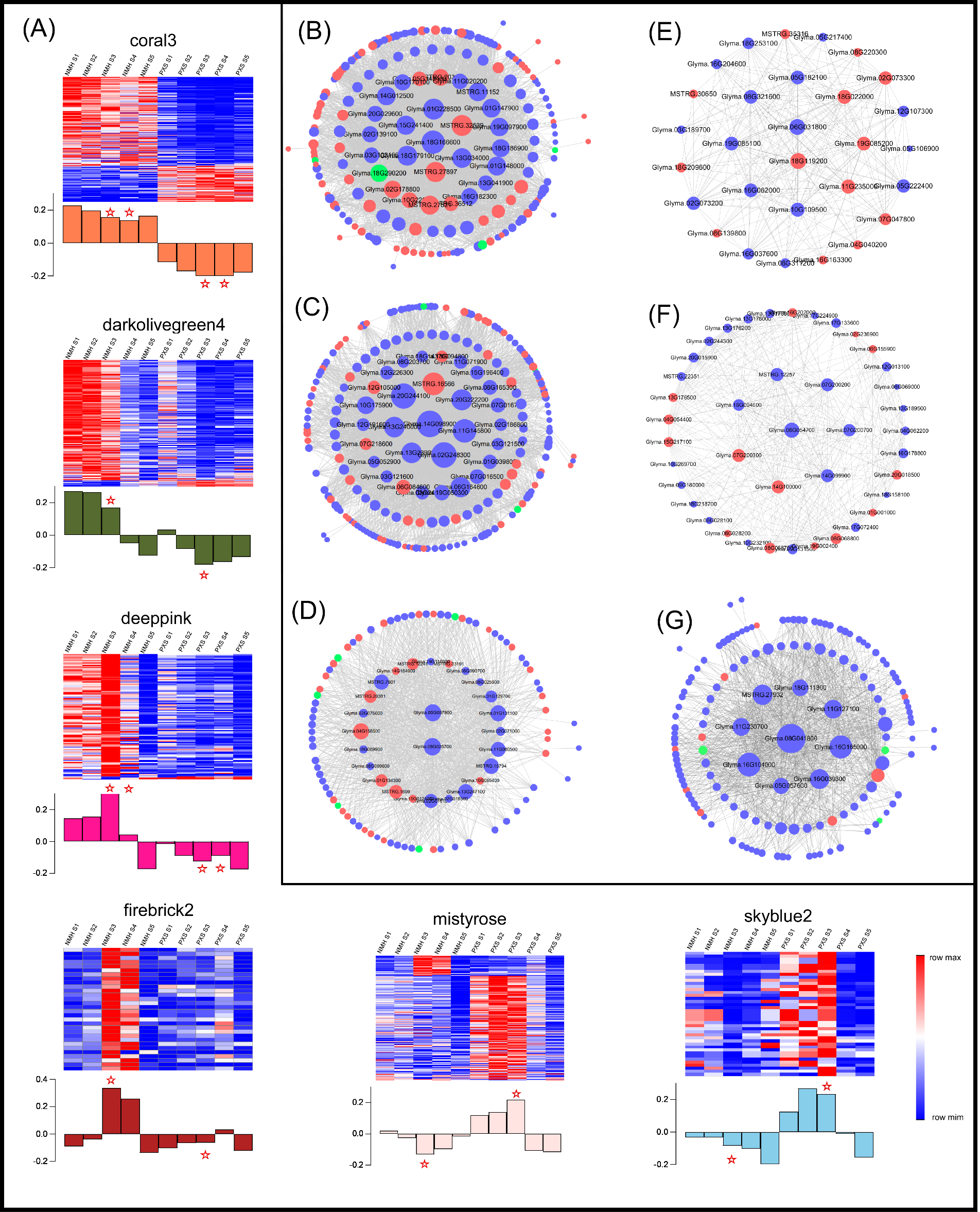


Fig. S5

Gene co-expression network maps and gene expression patterns of each module. (A) Heatmaps show the expression pattern of all the co-expressed genes in each module (red color represents a high level of expression, and blue color represents a low level of expression). The histograms show the variation in module eigengenes expressed in different samples. The stages with opposite expression patterns in NMH and PXS at Stages S3 or S4 have been marked with asterisks. (B-G) Show the gene co-expression network maps of the coral3 (B), darkolivegreen4 (C), deeppink (D), firebrick2 (E), mistyrose (F) and skyblue2 (G) modules, respectively. Node size represents gene connectivity, and node color represents gene category. Green indicates a transcription factor; blue represent a non-transcription factor; red refers to a gene that has not been clearly defined. The candidate hub genes are located at the center of each module.


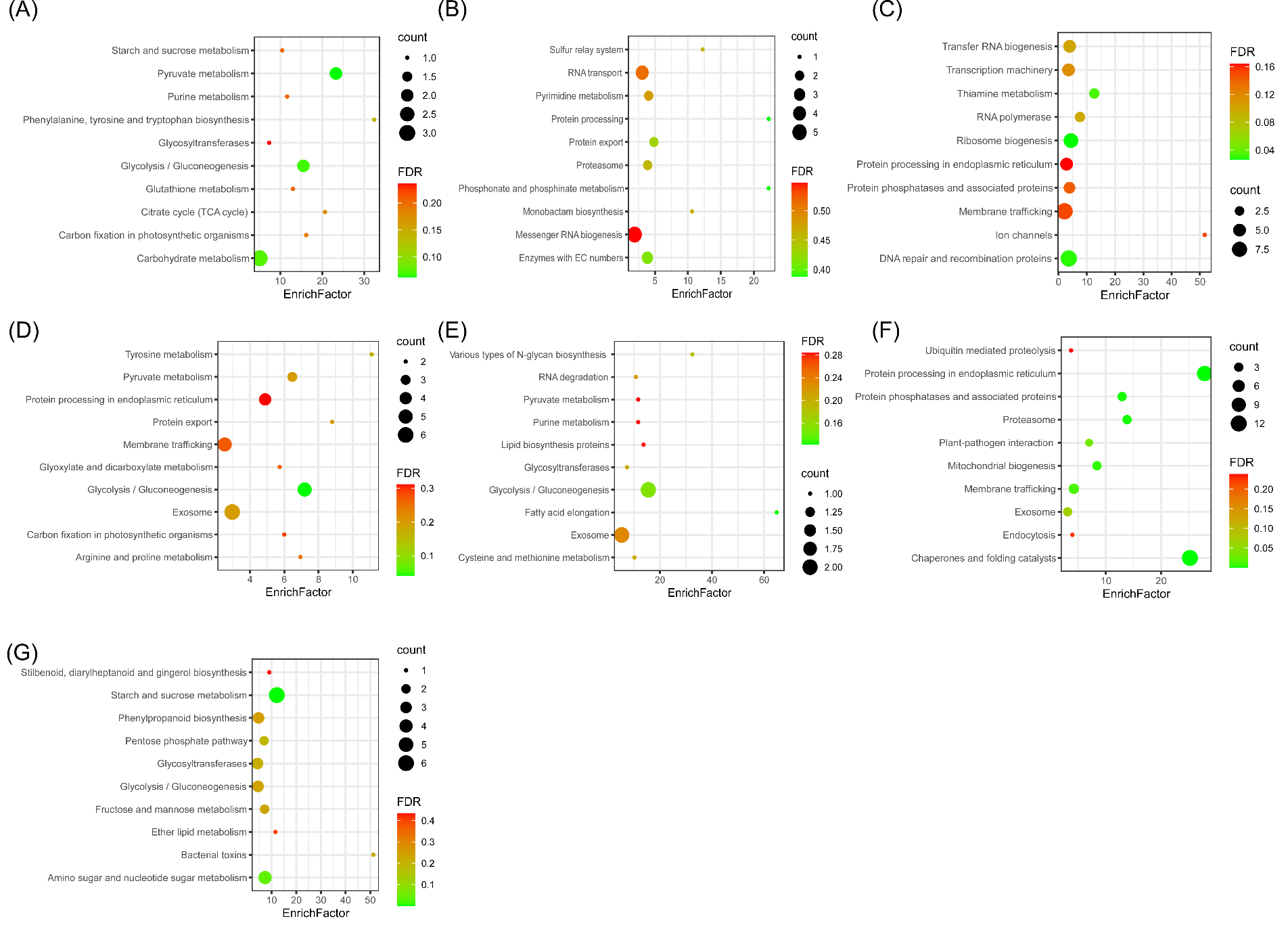


Fig. S6

KEGG pathway enrichment analysis of genes in seven modules. The top 20 KEGG pathway with the highest significance of antiquewhite1 (A), coral3 (B), darkolivegreen4 (C), deeppink (D), firebrick2 (E), mistyrose (F), and skyblue2 (G) modules.


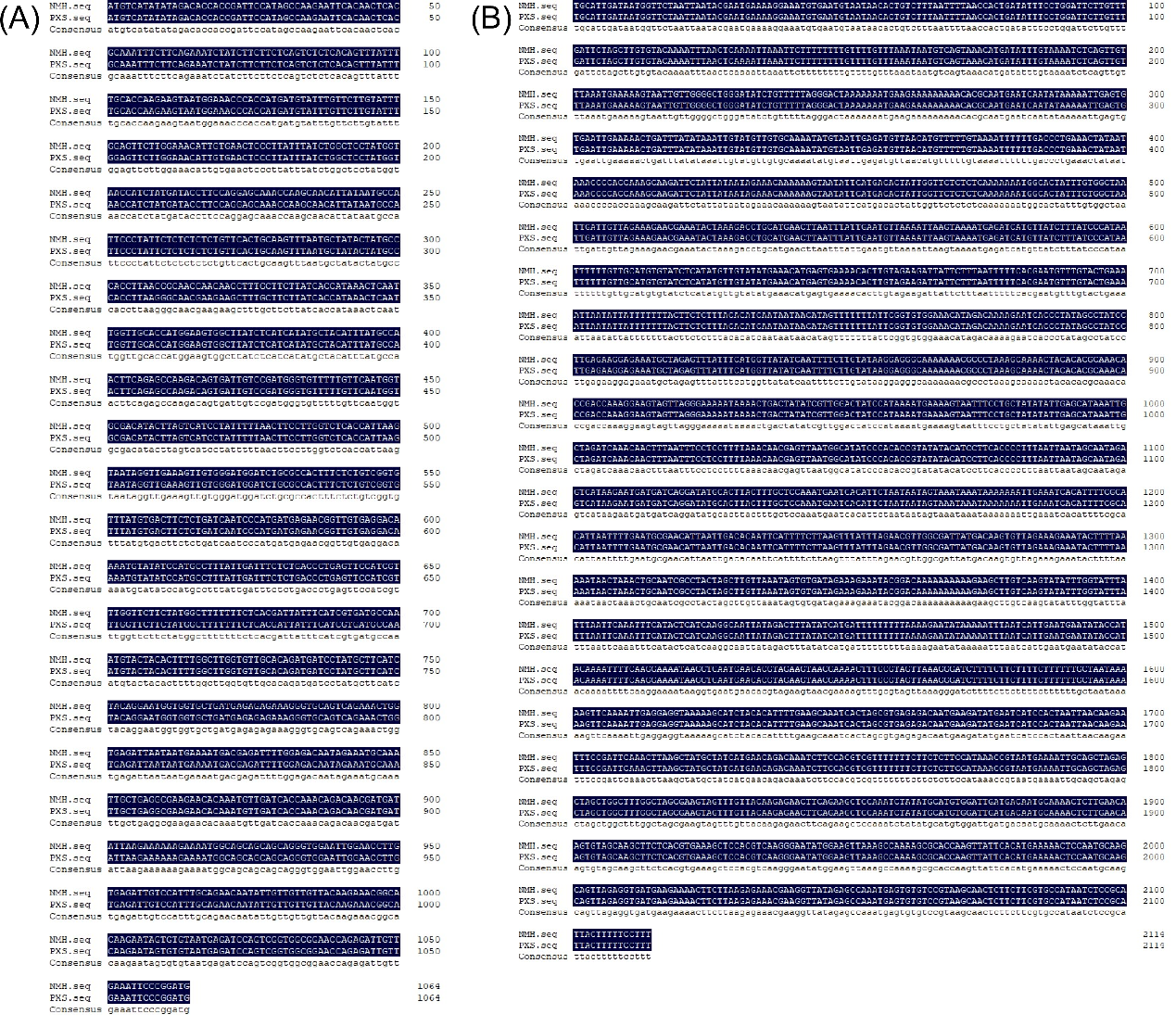


Fig. S7

The coding DNA sequences (A) and promoter sequences (B) of G*mSWEET2* from NMH and PXS


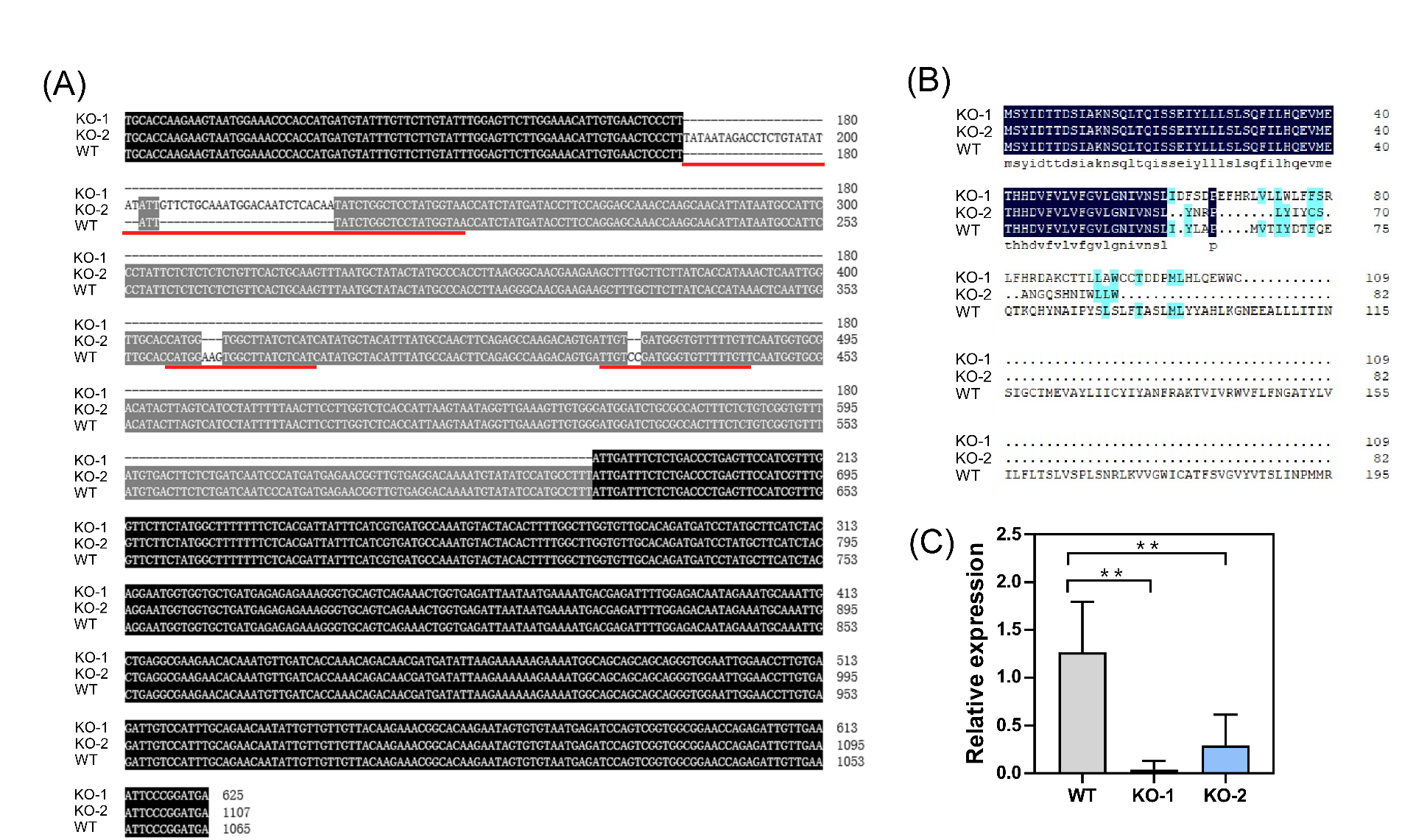


Fig. S8

Identification of *GmSWEET2*-knockout plants. (A) The DNA sequence of *GmSWEET2* wild type and two genome editing types at the target site (red underline). (B) The amino acid sequences of *GmSWEET2* wild type and two genome editing types. (C) The relative expression level of *GmSWEET2* in WT (NMH) and *GmSWEET2*-KO lines.


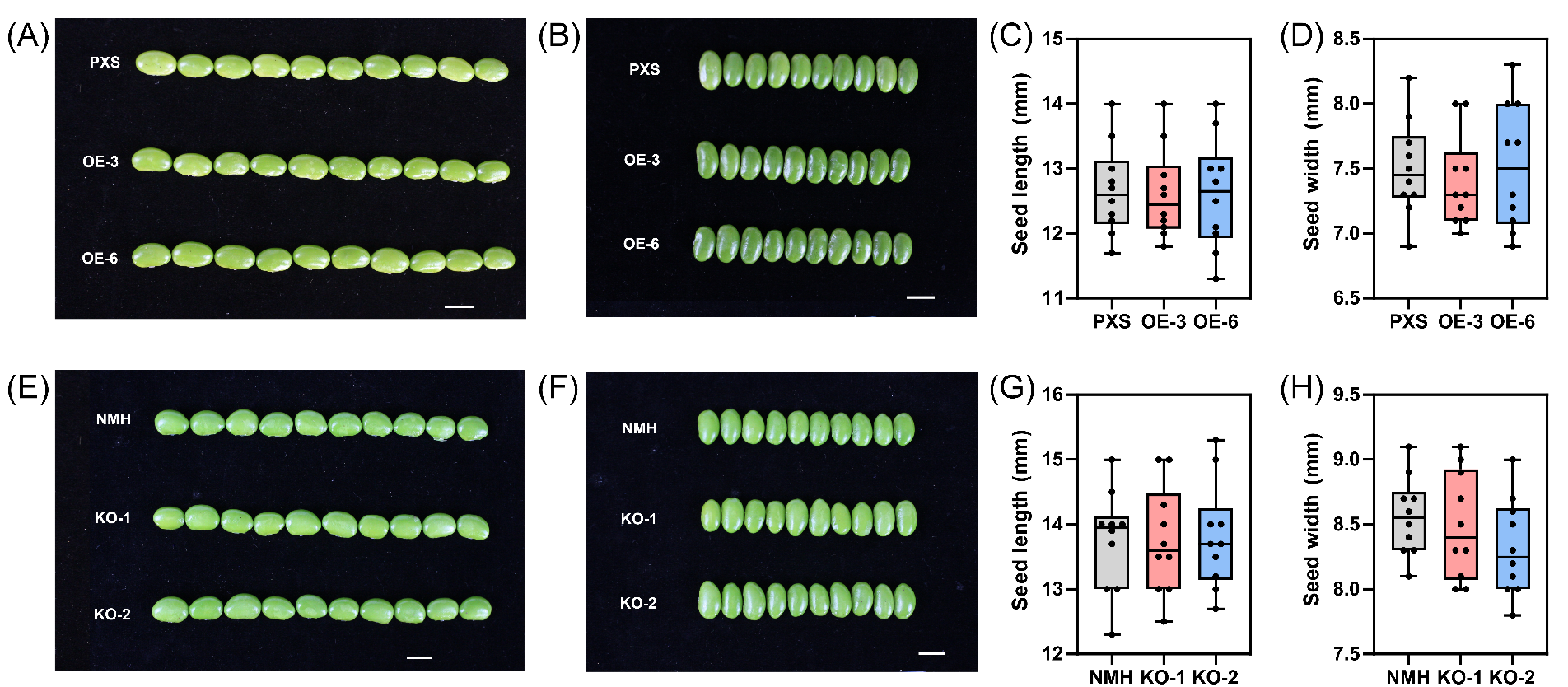


Fig. S9

The seed size of *GmSWEET2*-OE lines and *GmSWEET2*-KO lines. (A and B) Seeds of WT (PXS) and *GmSWEET2*-OE lines at Stages R6. (C and D) Seed dimensions of WT (PXS) and *GmSWEET2*-OE lines at Stages R6. Data are presented as mean ± SD (*n* = 10 biological replicates). **P* < 0.05, ***P* < 0.01 compared with PXS, Student’s *t* test. (E and F) Seeds of WT (NMH) and *GmSWEET2*-KO lines at Stages R6. (G and H) Seed dimensions of WT (NMH) and *GmSWEET2*-KO lines at Stages R6. Data are presented as mean ± SD (*n* = 10 biological replicates). **P* < 0.05, ***P* < 0.01 compared with NMH, Student’s *t* test.


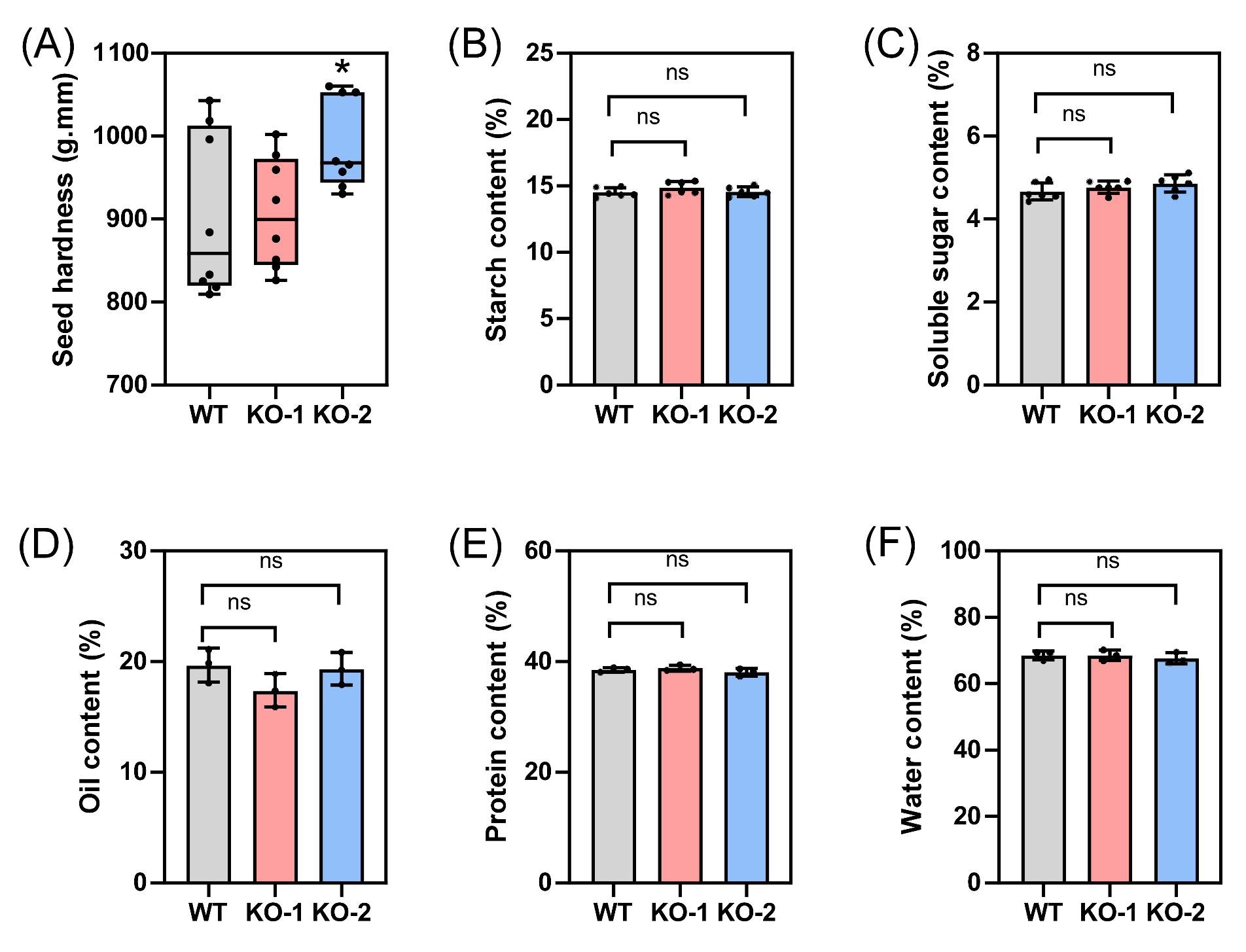


Fig. S10

Seed hardness and component contents of WT (NMH) and *GmSWEET2*-KO lines at Stage R6. (A) Seed hardness of WT (NMH) and *GmSWEET2*-KO lines at Stage R6. The box plot shows median (horizontal line) and individual values (black dots) (*n* = 8 biological replicates). **P* < 0.05, ***P* < 0.01 compared with WT, Student’s *t* test. (B-F) Starch, soluble sugar, oil, protein and water contents in WT (NMH) and *GmSWEET2*-KO lines at Stage R6; data are presented as mean ± SD [*n* = 6 for (B) and (C), *n* = 3 for (D) to (F)]. ns, no significant difference. **P* < 0.05, ***P* < 0.01, Student’s *t*-test.
